# Azithromycin augments rhinovirus-induced IFNβ via cytosolic MDA5 in experimental models of asthma exacerbation

**DOI:** 10.18632/oncotarget.16364

**Published:** 2017-03-18

**Authors:** Mandy Menzel, Hamid Akbarshahi, Ellen Tufvesson, Carl Persson, Leif Bjermer, Lena Uller

**Affiliations:** ^1^ Respiratory Immunopharmacology, Department of Experimental Medical Science, Lund University, 22184 Lund, Sweden; ^2^ Respiratory Medicine and Allergology, Department of Clinical Sciences Lund, Lund University, 22184 Lund, Sweden; ^3^ Division of Clinical Chemistry and Pharmacology, Department of Laboratory Medicine, Lund University, 22184 Lund, Sweden

**Keywords:** asthma, rhinovirus, azithromycin, MDA5, IFNβ

## Abstract

Deficient production of anti-viral interferons (IFNs) may be involved in causing viral-induced asthma exacerbations. Hence, drugs inducing lung IFN production would be warranted. Azithromycin may reduce asthma exacerbations but its modus operandi is unknown. Here, we investigated if azithromycin induces IFNβ expression *in vitro* in rhinovirus-infected bronchial epithelial cells from asthmatic donors and *in vivo* in our allergic inflammation-based mouse model of viral stimulus-induced asthma exacerbations. Azithromycin dose-dependently augmented viral-induced IFNβ expression in asthmatic, but not in healthy bronchial epithelial cells. The effect negatively correlated with viral load. Knockdown of MDA5 and RIG-I by siRNA showed involvement of MDA5 but not RIG-I in azithromycin's IFN-inducing effects *in vitro*. *In vivo* azithromycin induced IFNβ protein, restoring a reduced lung IFN response exclusively in allergic exacerbating mice. This was associated with induction of interferon-stimulated genes and MDA5, but not RIG-I. We suggest that clinically relevant concentrations of azithromycin produce MDA5-dependent, anti-viral, IFN-inducing effects in bronchial epithelium distinctly from asthmatic donors. Similarly, azithromycin induced MDA5-associated IFN in virally stimulated lungs *in vivo* exclusively in allergic mice. Effects of azithromycin and MDA5-active drugs on viral-induced exacerbations deserve further research.

## INTRODUCTION

For more than 150 years episodes of severe asthma exacerbations have been associated with common cold [1]. Modern PCR techniques demonstrate that about 80% of asthma exacerbations are triggered by infections with rhinoviruses [2]. The exacerbations are transient but may be causatively involved in worsening of asthma symptoms [3]. Mainstay therapy involving bronchodilators and inhaled corticosteroids provides relief to patients with stable asthma but is insufficient during exacerbations and in severe disease [4], which accounts for increasing healthcare cost spending in asthma [5].

Rhinoviruses infect both upper airways and bronchial epithelium. A viral replication intermediate – dsRNA – is sensed by pattern recognition receptors, Toll-like receptor 3 (TLR3) or the RIG-I like helicases melanoma differentiation-associated protein 5 (MDA5) and retinoic acid-inducible gene 1 (RIG-I) [6, 7]. This interaction leads to downstream production of interferons (IFNs) that are important anti-viral proteins regulating both innate and adaptive immunity. Several studies postulate a deficiency of anti-viral type I IFN production in asthmatics at rhinovirus infection [8, 9], which is correlated with poor viral clearance. Exogenous IFNβ has been shown to suppress rhinovirus infection *in vitro* in bronchial epithelial cells [10] and clinical trials on the use of IFNβ as a prophylactic drug in asthma [11] attract interest. Understanding mechanisms involved in bronchial epithelial IFNβ production and its pharmacological control in asthma emerges as an important area of investigation with a potential of discovering novel treatments for asthma exacerbations.

Azithromycin, a macrolide antibiotic, in standard dose regimens (producing *in vivo* levels of up to 10 μg/ml [12]) has produced promising effects on exacerbations of asthma [13]. Hence, it was of interest to explore anti-viral effects of clinically relevant concentrations of azithromycin in bronchial epithelial cells from asthmatic patients. It is hazardous to predict from findings in cell culture experiments what drug effects will occur in the dynamic, blood-perfused and innervated lungs. Hence, *in vivo* model studies are warranted.

Here, we employed a house dust mite (HDM)-based mouse model where an added viral stimulus induces reproducible asthma-like exacerbations [14]. Several aspects were considered for the choice of an *in vivo* exacerbation mouse model for exploring effects of azithromycin on pulmonary IFNβ response to viral-like stimulation. The basic allergic inflammation model of asthma should preferably involve a human asthma relevant allergen such as HDM that produces lung eosinophilic inflammation without the need of an adjuvant. Studies employing HDM and RV1B infection have been carried out but, as reported by Rochlitzer, Hoymann [15], no exacerbation could be detected. As an alternative poly(I:C) has been forwarded as an agent that mimics biological effects of rhinovirus infection [16] and has been successfully employed to produce exacerbation effects in mice with established HDM-induced pulmonary inflammation [14, 17]. Thus, increased eosinophilic and neutrophilic inflammation was induced together with general signs of inflammation including increased BALF levels of LDH [14], a typical feature of viral-induced human asthma exacerbations [18]. We hypothesized that HDM and poly(I:C) would produce an exacerbation where reduced IFNβ was produced and where it would be of particular interest to study potential IFNβ-promoting actions *in vivo* by intervention with azithromycin.

We demonstrate that azithromycin concentration-dependently augments viral infection-induced IFNβ expression in primary bronchial epithelial cells from asthmatic distinct from healthy donors. At low and clinically relevant concentrations [12], this drug action further correlates negatively with viral load. *In vivo* azithromycin amplifies IFNβ expression in allergic but not in non-allergic mice stimulated with a viral mimic. Furthermore, we demonstrate that MDA5 is involved in azithromycin's IFN-inducing effects *in vitro* and *in vivo*.

## RESULTS

### Azithromycin augments rhinovirus-induced IFNβ expression in primary bronchial epithelial cells from asthmatics, which is associated with over-expression of RIG-I like receptors and repression of viral replication

**Figure 1:**
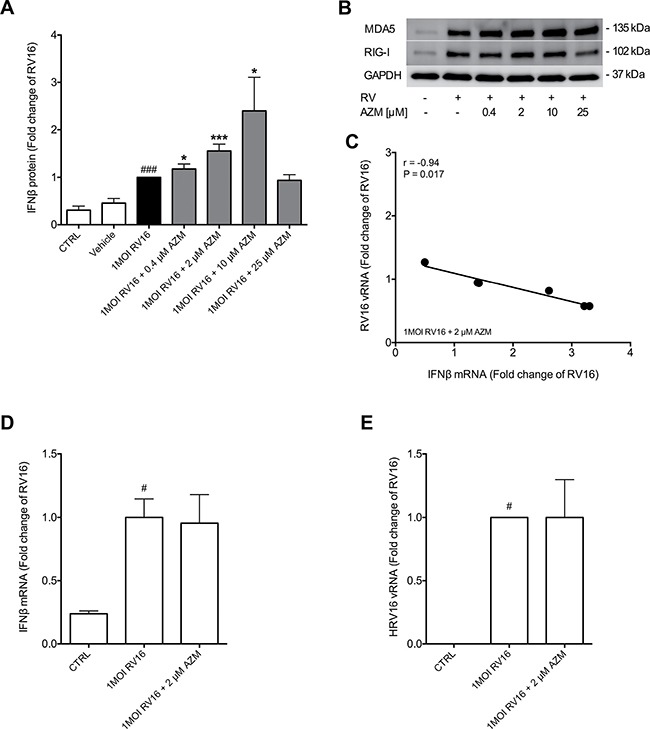
Azithromycin (AZM) augments rhinovirus-induced expression of IFNβ and RIG-I like helicases, while repressing viral infection exclusively in primary bronchial epithelial cells from asthmatic donors HBECs from asthma patients and healthy individuals were pre-treated with azithromycin for 24 h before infection with 1MOI RV16 and continuous throughout the experiment. Cells were harvested for gene and protein expression analysis 24 h and 48 h post infection, respectively. Protein expression levels of IFNβ **(A)** were measured by ELISA and gene expression levels of IFNβ **(D)** and viral load **(E)** were measured by real-time PCR. Data is presented as mean ± standard error of the mean (SEM) fold change of RV16. Comparison of different groups was performed by Kruskal-Wallis with Wilcoxon post testing. ^#^*p* < 0.05, ^##^*p* < 0.01, ^###^*p* < 0.001 vs. CTRL; *p < 0.05, **p < 0.01, ***p < 0.001 AZM vs. RV16. Data was obtained from 7 asthmatic and 4 healthy donors. A representative Western Blot image of MDA5 and RIG-I protein is shown **(B)**. Correlation analysis between IFNβ and HRV16 mRNA was performed **(C)**. Correlations were analysed by Spearman. For correlation with a *P-value* below 0.05, and thus regarded statistically significant, linear regression was employed.

Infection of primary bronchial epithelial cells from asthmatic subjects with 1MOI RV16 induced release of IFNβ into cell supernatants 48 h post infection (*p* < 0.001; Figure 1A). Treatment with azithromycin in different concentrations beginning 24h before infection with rhinovirus and continuous throughout the experiment significantly augmented RV-induced IFNβ secretion in a concentration-dependent manner (Figure 1A), while azithromycin treatment alone did not stimulate IFNβ secretion (data not shown). This was associated with a slight enhancement of RV-stimulated expression of the RIG-I like helicases RIG-I and MDA5 by azithromycin (Figure 1B). Exogenous IFN administration has been shown to suppress viral replication *in vitro* [10]. To determine if azithromycin-induced IFNβ expression was able to repress viral replication, a correlation analysis between IFNβ mRNA and viral load for a low IFN-stimulating dose of 2 μM azithromycin was performed. Individual expression of IFNβ correlated negatively with viral load, suggesting that azithromycin-induced IFNβ expression might be sufficient to impair viral replication (*p* < 0.05; Figure 1C).

### Azithromycin does not alter viral-induced IFNβ expression in primary bronchial epithelial cells from healthy individuals

In order to determine if azithromycin's IFN-inducing effect and thus its viral load-reducing properties are limited to diseased epithelium, bronchial epithelial cells from healthy subjects were treated with 2 μM azithromycin before and during infection with RV16. Infection with RV16 significantly induced IFNβ expression in healthy epithelium (*p* < 0.05). However, treatment with azithromycin did not alter viral-induced IFNβ expression in these cells (Figure 1D). Further, azithromycin did not affect viral replication in the healthy bronchial epithelial cells (Figure 1E).

### Asthma-like exacerbations in mice

**Figure 2:**
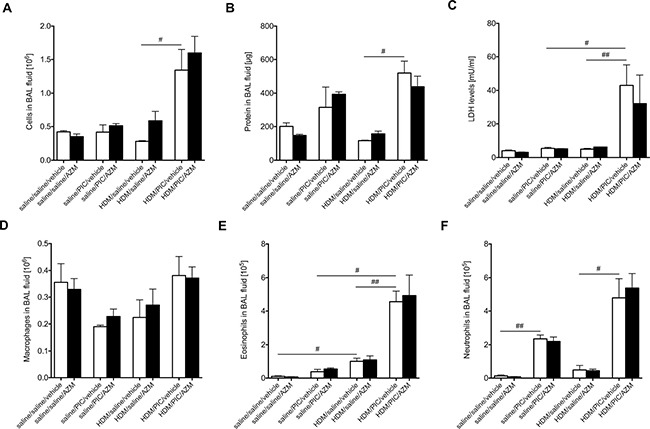
No effect of azithromycin (AZM) on bronchoalveolar lavage inflammatory parameters and LDH levels in a mouse model of asthma exacerbation Mice were challenged with HDM or saline 3 days/week for 3 weeks and were subsequently exposed daily for 3 days to poly(I:C) or saline. Mice were treated once daily with azithromycin or vehicle starting 48h before and continuing throughout the poly(I:C) challenges. Lavage of the lungs was performed with PBS 24 h after the final poly(I:C) exposure. Cell count **(A)**, protein concentration **(B)**, LDH levels **(C)**, macrophages **(D)**, eosinophils **(E)** and neutrophils **(F)** in BAL fluid were measured and data is presented as mean ± standard error of the mean (SEM). Comparison of different groups was performed by Kruskal-Wallis with Wilcoxon post testing. ^#^*p* < 0.05, ^##^*p* < 0.01. 4-9 mice per group.

Azithromycin showed a consistent IFN-inducing effect in the present viral-infected diseased bronchial epithelial cells *in vitro*. In order to assess if azithromycin also exhibits IFN-inducing properties in an *in vivo* system, with additional dynamic complexity involving humoral and neural mechanisms, we selected to use a mouse model of asthma exacerbation that we have developed [14]. Asthma-like exacerbations were thus produced by a sequential combination involving HDM-induced lung inflammation followed by stimulation with the TLR3 agonist poly(I:C). The combined HDM and poly(I:C) challenges induced a significant increase in influx of inflammatory cells, total protein and lactate dehydrogenase (LDH) into the airways as picked up by bronchoalveolar lavage fluid (BALF; Figure 2A–2C). There was a significant increase in eosinophilia in HDM-challenged mice and induction of neutrophils in poly(I:C)-stimulated mice. In mice receiving both HDM and poly(I:C) both neutrophils and eosinophils were further elevated (Figure 2E and 2F).

### Azithromycin induces neither general inflammatory parameters nor LDH release in a mouse model of asthma exacerbation

There was no significant difference in the general cell and protein inflammatory parameters between mice treated either with vehicle or azithromycin (Figure 2A and 2B). To investigate if azithromycin treatment induced any unwanted cell death, levels of the general necrosis marker LDH were measured in BALF and, again, no difference between vehicle-treated and azithromycin-treated animals was observed (Figure 2C). Further, azithromycin treatment did not alter the composition of recruited inflammatory cells (Figure 2D–2F).

### Deficient IFNβ production in our *in vivo* model of asthma exacerbations – restoration by azithromycin

**Figure 3:**
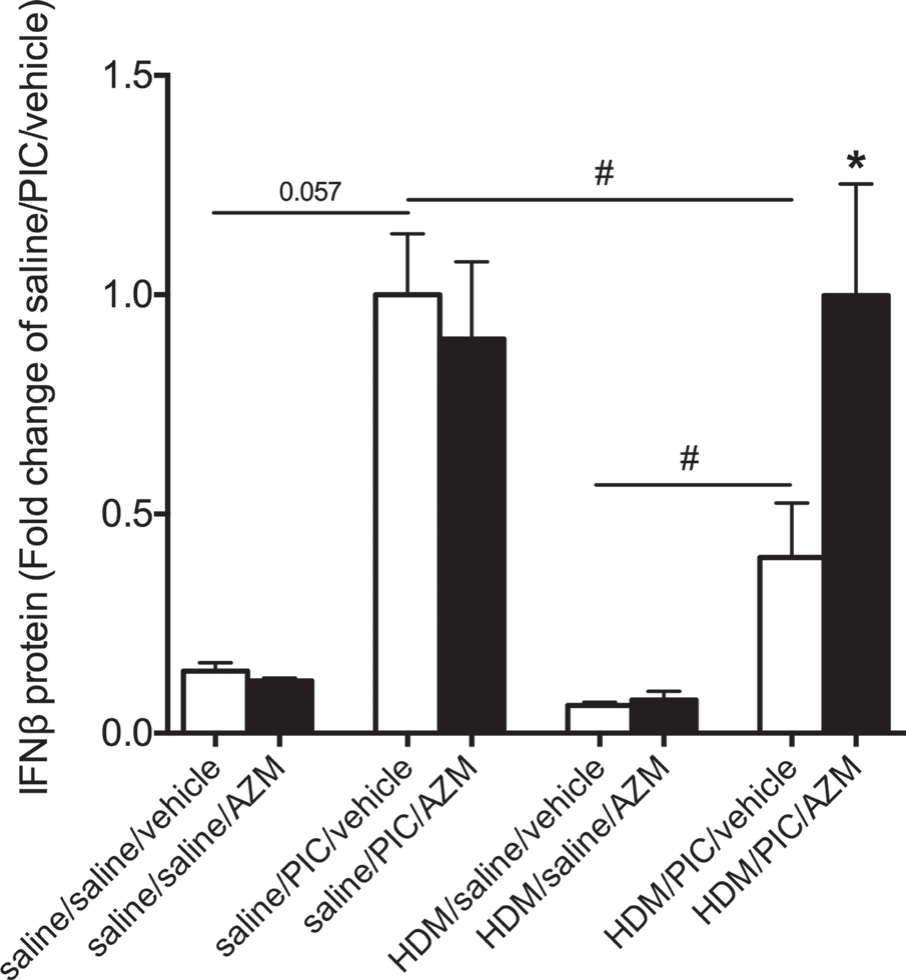
Azithromycin (AZM) augments expression of IFNβ *in vivo* at viral stimulus-induced asthma exacerbations Mice were challenged with HDM or saline 3 days/week for 3 weeks and were subsequently exposed daily for 3 days to poly(I:C) or saline. Mice were treated once daily with azithromycin or vehicle starting 48 h before and continuing throughout the poly(I:C) challenges. Lavage of the lungs was performed with PBS 24h after the final poly(I:C) exposure. Protein expression levels of IFNβ were measured by ELISA and data is presented as mean ± standard error of the mean (SEM). Comparison of different groups was performed by Kruskal-Wallis with Wilcoxon post testing. #*p* < 0.05; *p < 0.05 vehicle vs. AZM. 4-9 mice per group.

The notion that asthmatics exhibit a deficient IFN production has attracted interest as a favored explanation of the particularly severe consequences of common cold in these patients. Importantly, therefore, we observed that our exacerbation model exhibited a significantly diminished IFNβ response to viral stimulation compared to animals where viral stimulation was introduced without a background of allergic inflammation (Figure 3). Next we wanted to investigate if azithromycin was able to induce IFNβ expression in an *in vivo* setting. In our model, exposure to poly(I:C) induced IFNβ levels (*p* = 0.057; Figure 3), which was significantly diminished in mice challenged with HDM prior to exposure to poly(I:C). While azithromycin did not further induce poly(I:C)-induced IFNβ levels in non-allergic mice, it augmented – and thus restored – IFNβ levels in allergic mice stimulated with poly(I:C) (*p* < 0.05; Figure 3). This distinction is similar to our *in vitro* findings where azithromycin augmented RV-induced IFN expression in bronchial epithelial cells from asthmatics (Figure 1A) but not in epithelium from healthy individuals (Figure 1D).

### Azithromycin augments expression of interferon-stimulated genes and the pattern recognition receptor MDA5 but not RIG-I in exacerbating mice

**Figure 4:**
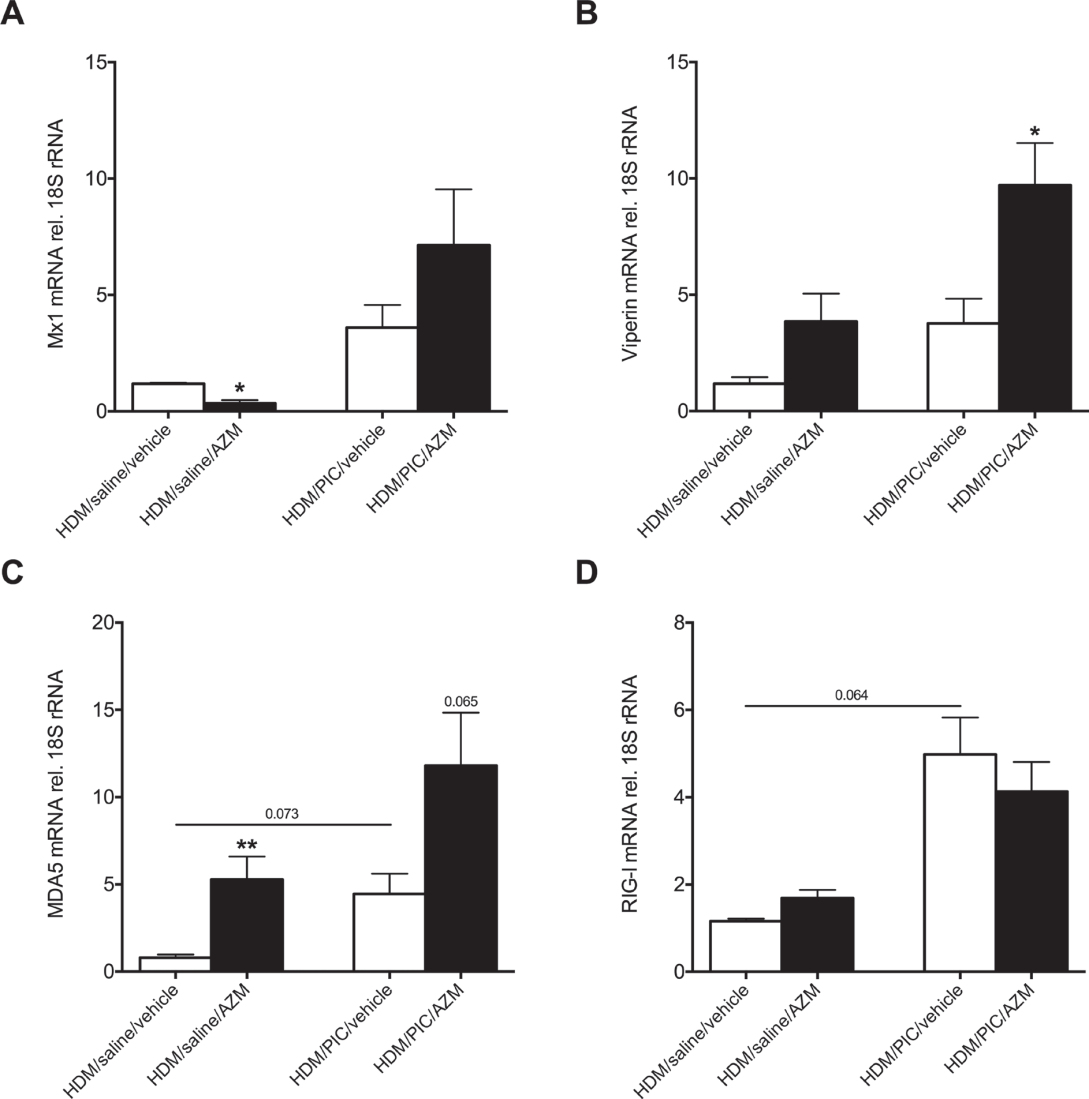
Azithromycin (AZM) augments expression of interferon-stimulated genes and the pattern recognition receptor MDA5 but not RIG-I in lung homogenates of exacerbating mice Mice were challenged with HDM 3 days/week for 3 weeks and were subsequently exposed daily for 3 days to poly(I:C) or saline. Mice were treated once daily with azithromycin or vehicle starting 48h before and continuing throughout the poly(I:C) challenges. Gene expression levels of Mx1 (**A**), viperin (**B**), MDA5 (**C**) and RIG-I (**D**) were measured in lung homogenates by real-time PCR and data is presented as mean ± standard error of the mean (SEM) fold change of HDM/saline/vehicle relative to 18S rRNA expression. Comparison of different groups was performed by Kruskal-Wallis with Wilcoxon post testing. *p < 0.05, **p < 0.01 vehicle vs. AZM. 5-9 mice per group.

We further investigated if genes downstream and upstream of IFNβ expression were altered by azithromycin treatment in exacerbating mice. Mx1 and viperin are interferon-stimulated genes displaying anti-viral properties [19, 20]. There was a slight induction of Mx1 (Figure 4A) and a significant increase in viperin lung expression (*p* < 0.05; Figure 4B) in exacerbating mice after azithromycin treatment compared to vehicle control. Viral dsRNA is recognized by pattern recognition receptors, like the RIG-I like helicases MDA5 and RIG-I, which leads to activation of a signalling cascade in which end IFN expression is initiated. While expression of lung MDA5 tended to be elevated by treatment with azithromycin in the exacerbation group (*p* = 0.065; Figure 4C), azithromycin did not alter lung RIG-I expression (Figure 4D), suggesting that these pattern recognition receptors may have differing roles in azithromycin's induction of IFN expression.

### Knockdown of MDA5, but not knockdown of RIG-I, diminishes azithromycin-enhanced viral-induced IFNβ expression in asthmatic primary bronchial epithelial cells

**Figure 5:**
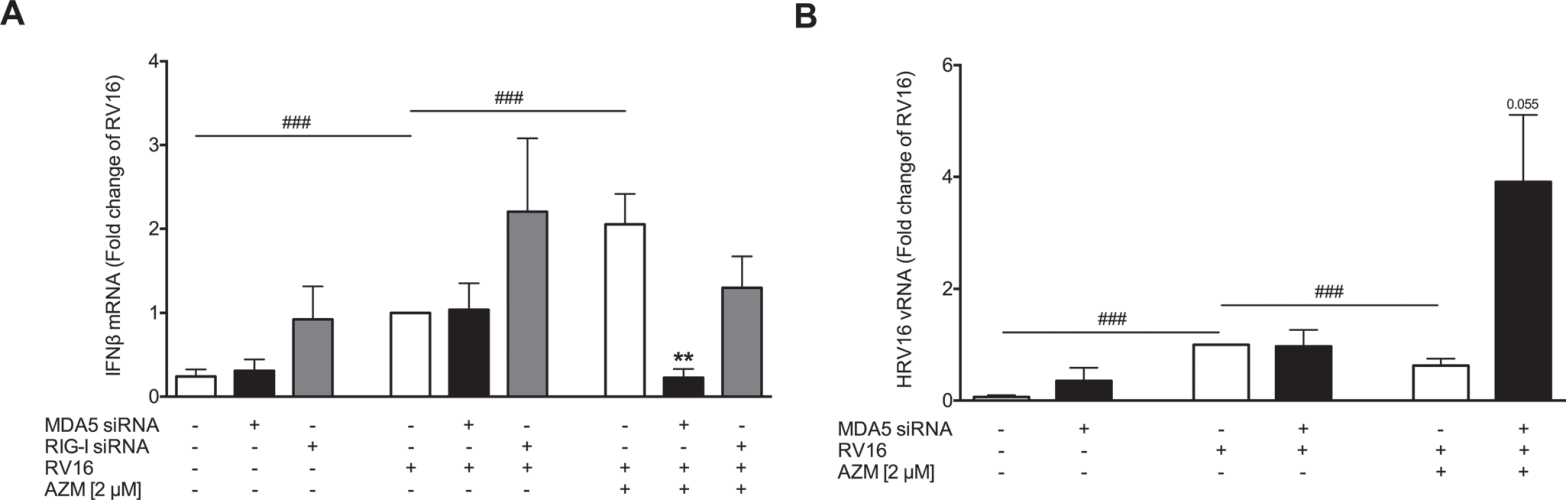
Effect of RIG-I like helicases knockdown on azithromycin (AZM)-augmented RV-induced expression of IFNβ and reduction of viral load in primary bronchial epithelial cells from asthmatic donors HBECs from asthmatic patients were transfected with siRNA specific for MDA5 or RIG-I and were then treated with azithromycin for 24 h before infection with 1MOI RV16 and continuous throughout the experiment. Cells were harvested for gene expression analysis 24 h post infection. Gene expression of IFNβ **(A)** and viral load **(B)** were measured by real-time PCR and data is presented as mean ± standard error of the mean (SEM) fold change of RV16 relative to UBC/GAPDH expression. Comparison of different groups was performed by Wilcoxon. ^#^*p* < 0.05, ^##^*p* < 0.01, ^###^*p* < 0.001; *p < 0.05, **p < 0.01 vs. corresponding control. Data was obtained from 5 donors.

To further elucidate if azithromycin's IFN-inducing effect was dependent on induction of either RIG-I like helicase, MDA5 and RIG-I were knocked down by siRNA specific for these pattern recognition receptors in primary bronchial epithelial cells of asthmatic patients. For these experiments a dose of 2 μM azithromycin was selected since we previously demonstrated a correlation between IFNβ and MDA5 gene expression using this dose of azithromycin [21]. Transfection with specific siRNA achieved an almost complete knockdown of MDA5 and RIG-I expression, respectively (Supplementary Figure 1). While knockdown of either RIG-I like helicase individually had no effect on RV-induced IFNβ expression, knockdown of MDA5 significantly reduced IFNβ expression in RV-infected and azithromycin-treated primary bronchial epithelial cells from asthmatics (*p* < 0.01). By contrast, knockdown of RIG-I had no significant effect (Figure 5A). Knockdown of MDA5 also enhanced viral load in RV-infected and azithromycin-treated epithelium (Figure 5B), supporting a role of MDA5 and strengthening an association between lack of IFNβ expression and increase in viral load.

## DISCUSSION

In this study we have made several novel observations related to viral-induced exacerbations of asthma and its potential treatment. By the use of bronchial epithelial cells from asthmatic donors we first obtained data on drug-induced IFNβ production that could not be seen in healthy bronchial epithelium. Thus, we revealed IFNβ-inducing effects by low concentrations of azithromycin, a macrolide antibiotic, in asthmatic epithelium. These findings are of interest because they concern anti-viral drug opportunities targeting a main location for rhinovirus infections in asthma. Importantly, a positive IFNβ effect by azithromycin was then discovered in our *in vivo* animal model of exacerbations that exhibits similarities to human asthma including a reduced lung production of IFNβ in response to viral stimulation. The present azithromycin-induced enhancement of viral stimulus-induced IFN production required a baseline of disease expressed as allergic inflammation in mice and asthma origin of bronchial epithelial cells in human. Furthermore, our *in vitro* and *in vivo* data jointly suggests the possibility that MDA5 receptors are involved in azithromycin's IFN-inducing actions.

Clinical studies have been employed using the macrolide azithromycin for prevention of asthma exacerbations. However, the benefit of macrolide treatment has so far been inconclusive [22]. Asthma is a heterogeneous disease with many phenotypes [23], thus hampering discovery of novel treatment opportunities, which would be beneficial in asthma patients with altered anti-viral responses to viral infections. While some studies propose that cells of asthmatics have a deficient IFN response towards experimental viral infections [8, 9], other studies could not confirm these findings [24]. Both differences in experimental setup between the studies and differences in asthma phenotype of the study subjects may have contributed to the different observations. Hence, a treatment that boosts anti-viral responses might not be suitable for all asthma patients. This notion agrees with the results of a recent clinical trial [13] in which azithromycin only produced beneficial effects in reducing exacerbation frequency in a subgroup of severe asthma with low blood eosinophilia (≤ 200/μl) where lung neutrophilia would be expected to occur. The present *in vivo* exacerbation model where azithromycin increased airway levels of IFNβ was also associated with pronounced lung neutrophilia [14].

A clinical trial aiming at reducing viral-induced asthma exacerbations using exogenous IFNβ therapy showed a trend towards reducing viral load [11]. Supporting the possibility of efficacy of this treatment, *in vitro* administration of IFNβ had been shown to inhibit rhinoviral replication in bronchial epithelial cells [10]. In the present primary bronchial epithelial cells from asthmatic patients azithromycin treatment augmented viral infection-evoked IFNβ production and the effect was dose-dependent. Further, our correlation analysis demonstrated that, already at a low dose, azithromycin-induced IFNβ expression was negatively correlated with viral load. The effect was produced in the same concentration range as was efficacious in COPD cells [21]. The present findings thus indicate a similarity between asthmatic and COPD epithelium with regard to IFN responsiveness to azithromycin. This is of interest because rhinovirus infection is a major cause of difficult-to treat asthma as well as COPD exacerbations and azithromycin has shown promising effects by reducing exacerbation frequency and improving quality of life in COPD [25]. While asthma and COPD are both chronic inflammatory diseases, they differ in their nature of inflammation, with asthma considered predominantly eosinophilic, while COPD is characterised by neutrophilic inflammation [26]. However, during viral-induced exacerbation there is an influx of neutrophils in asthmatics and a rise in eosinophilia in COPD patients, making the inflammatory characteristics of both diseases more similar [27]. In this context it is of interest that the present *in vivo* exacerbation model exhibited mixed granulocyte inflammation.

Interestingly, in healthy bronchial epithelium azithromycin did not display any IFN-inducing properties and, in accord, did not alter viral load. In accord with the present observation, we have previously demonstrated lack of IFN-inducing effect of azithromycin in healthy bronchial epithelial cells that were obtained from a different cohort of healthy donors [21]. Our repeated findings in this regard are at variance with an early report on azithromycin-induced IFN expression and anti-viral signalling in bronchial epithelium from healthy individuals [28]. The effective concentrations in that study [28] were thus more than a magnitude higher than effective drug levels in diseased epithelium in this study. We have therefore not pursued the use of high concentrations of azithromycin to potentially be able to explain differing data between the different laboratories regarding effects of azithromycin in bronchial epithelium from healthy donors.

The present cell culture experiments were performed as submersion cultures rather than air-liquid-interface cultures where differentiated epithelial cells occur. However, our approach can also be seen as a potential asset. In submersion cultures the epithelial cells maintain basal cell-like undifferentiated properties that are more susceptible to rhinovirus infections than suprabasal cells [29].

Information regarding azithromycin's potential anti-viral and anti-asthma effects in the complex and dynamic *in vivo* milieu is scarce. Hence, animal model approaches could contribute to understanding its mode of action. Models of severe and exacerbating asthma are of particular interest because they represent areas where the need for novel treatments is great. For this study we employed a mouse asthma exacerbation model that we have developed involving a baseline of HDM-induced allergic airway inflammation that is markedly aggravated by additional challenges with a viral stimulus [14]. We used the TLR3 agonist poly(I:C) that mimics biological effects of rhinovirus infection. This approach was preferred because previous studies involving HDM-induced inflammation followed by RV1B infection in mice have failed to produce exacerbation [15]. Combining HDM and other types of virus infection than rhinovirus has produced important but variable data on exacerbation with and without eosinophilia [30, 31]. Also, IFNβ has not been detected in these studies [31]. Here, we achieved a mixed eosinophilic-neutrophilic lung inflammation phenotype with similarities to severe asthma [32]. Furthermore, we obtained robust exacerbation responses. We first demonstrated that mice receiving HDM-challenges responded with reduced induction of IFNβ after exposure to poly(I:C) compared with unchallenged mice. This finding intriguingly agrees with previous *in vitro* reports in which bronchial epithelial cells from asthmatics show a deficient IFNβ response to poly(I:C) as well as to rhinovirus infection [8, 9, 33]. Interestingly, while azithromycin did not further enhance IFNβ expression in non-allergic mice exposed to poly(I:C) it did so in mice with established allergic inflammation prior to the poly(I:C) challenges. Hence, azithromycin was able to restore a deficient IFNβ response to levels occurring in non-allergic, dsRNA challenged mice. This data suggests the possibility of a particularly beneficial action of azithromycin in asthma patients with a deficient IFN response. Indeed, our observations both *in vitro* and *in vivo* suggest that the drug action requires disease conditions, supporting its possible treatment benefits during viral-induced asthma exacerbations.

Viral dsRNA induces IFNs by activation of pattern recognition receptors. Such pattern recognition receptors include TLR3 [6] and the RIG-I like helicases RIG-I and MDA5 [7]. In our mouse model, treatment with azithromycin augmented poly(I:C)-induced MDA5 expression in the exacerbation group but it did not alter poly(I:C)-induced RIG-I expression, suggesting that these two pattern recognition receptors may play different roles in azithromycin-induced IFN expression. To investigate further if azithromycin-induced IFNβ expression is dependent on activation of RIG-I and MDA5, we used siRNA directed against these pattern recognition receptors, respectively. Studies investigating the role of pattern recognition receptors to RV infection in bronchial epithelial cells have reported a requirement for TLR3 and MDA5 but differ whether [34] or not [35] RIG-I expression is necessary for type I IFN expression. In our experimental setup knockdown of neither MDA5 nor RIG-I had an effect on RV-induced IFNβ expression. This discrepancy could be due to differences in the rhinovirus serotype (RV16 vs. RV1B), the acquisition and type of epithelial cells (HBECs vs. Beas2B or tracheal epithelial cells) or differences in signalling mechanisms between healthy and diseased. As RIG-I siRNA showed a tendency to induce IFNβ gene expression, we cannot exclude that the epithelial cells sense this siRNA as an innate stimulus. Interestingly, knockdown of MDA5 nearly abolished azithromycin-augmented RV-induced IFNβ expression, which was associated with enhanced viral load, suggesting that azithromycin induces IFNβ in a MDA5 dependent manner, which is sufficient to reduce viral load.

In conclusion, we have demonstrated that clinically relevant concentrations of azithromycin increased IFNβ production in RV-infected bronchial epithelial cells from asthmatics but had no effect in cells from healthy donors. The present study importantly included *in vivo* approaches where we discovered first that mice with allergic inflammation exhibited an abnormally low IFNβ production in response to a viral stimulus and second that azithromycin restored the IFNβ response in these mice. Functional studies *in vitro* and association data *in vivo* indicated involvement of the pattern recognition receptor MDA5 in azithromycin's actions. The present data thus supports the possibility that azithromycin may restore deficient lung IFN production in exacerbating asthma and that this effect is MDA5-dependent. We also suggest that further studies are warranted to exploit the possibility that other molecules, sharing azithromycin's MDA5-dependent mechanism, may have a role in treatment of asthma exacerbations.

## MATERIALS AND METHODS

### Culturing of bronchial epithelial cells

Primary cultures of human bronchial epithelial cells (HBECs) from asthmatic donors were obtained by bronchoscopy using a fibre-optic bronchoscope (Olympus, IT160, Tokyo, Japan). For sampling of epithelial cells from bronchi standard sterile-sheared nylon cytology brushes were used. The procedure was performed in accordance with standard published ethical guidelines and was processed as described previously [33]. The patients showed no clinical signs of an infection. Written informed consent was obtained from all participants and the study was approved by the regional ethical review board at Lund University (permit no. 218/2011). A patient characterisation can be found in Table 1. HBECs were cultured in bronchial epithelial growth medium. In further experiments HBECs from 4 healthy individuals (Lonza, Walkersville, MD, USA) were utilized.

**Table 1:** Patient characteristics of the asthma patients included in this study

Gender	Age [years]	FEV_1_ [%pred]	Atopy	Concomitant medication
Male	41	81.1	Yes	ICS, SABA
Female	22	96.6	Yes	None
Female	24	101.1	Yes	ICS, LABA
Male	25	102.5	Yes	None
Female	19	115.9	Yes	ICS, SABA
Female	25	79.8	Yes	None
Female	25	96.3	Yes	None
Female	26	102.5	Yes	ICS, SABA
Female	32	101.5	Yes	ICS

Spirometry data was obtained without administration of bronchodilators.

FEV_1_, forced expiratory volume in 1s; ICS, inhaled corticosteroids; LABA, long-acting beta-adrenoceptor agonist; SABA, short-acting beta-adrenoceptor agonist.

HBECs were seeded into 12-well plates (Nunc, Life Technologies, Carlsbad, CA, USA) and experiments were initiated when the cell monolayer was 80-90% confluent. In all experiments cells were used at passage 2-3.

### Azithromycin treatment

Azithromycin (Sigma-Aldrich, Stockholm, Sweden) was dissolved in dimethyl sulfoxide (DMSO) and HBECs were exposed to azithromycin 24h prior to infection with rhinovirus and continuous throughout the experiment.

### Rhinovirus infection

The major group rhinovirus RV16 was grown in Ohio HeLa cells (European Collection of Cell Cultures) as previously described [36] and was obtained from clarified cell lysates. HBECs were infected with RV16 at 1 multiplicity of infection (MOI) [28, 37] for 1h at room temperature while shaking. Then the virus was removed and fresh BEGM medium containing azithromycin was added. Cell lysates and supernatants were obtained 24h and 48h post infection, respectively, and utilized for gene and protein expression analysis.

### Transfection with siRNA

HBECs were transfected with siRNA targeted against MDA5 (ID: 125361) or RIG-I (ID: s223616) or with non-specific siRNA (Ambion, Thermo Scientific, Waltham, MA, USA) in a concentration of 10 μM using Lipofectamine RNAiMAX (Ambion) as a transfection agent.

### Mouse model study design

All experiments were approved by the regional laboratory animal ethics committee in Malmö/Lund (permit no. M36-13) and were in accordance with standard published ethical guidelines. C57BL/6 mice were challenged with HDM (Greer, Lenoir, NC, USA) and were subsequently exposed to poly(I:C) (InvivoGen, San Diego, CA, USA) as previously described [14]. Mice were treated with 50 mg/kg azithromycin or vehicle 48h before and during poly(I:C) stimulation once daily. The experiment was terminated 24h after the last poly(I:C) exposure and bronchoalveolar lavage fluid and lung homogenates were collected.

### Statistical analysis

Data is presented as mean with standard error of the mean. Statistical analysis was performed in R [38]. *P*-values < 0.05 were regarded statistically significant. In brief, statistical significance of multiple comparisons was determined by Kruskal-Wallis test with Wilcoxon signed-rank post test to investigate differences between two groups in multiple selections. For single selections only Wilcoxon signed-rank test was used. Spearman's correlation was employed for correlation analysis.

## SUPPLEMENTARY MATERIALS FIGURES



## Abbreviations

BALF: bronchoalveolar lavage fluid
COPD: chronic obstructive pulmonary disease
HBEC: human bronchial epithelial cell
HDM: house dust mite
IFNβ: interferon-beta
LDH: lactate dehydrogenase
MDA5: melanoma differentiation-associated protein 5
Mx1: interferon-induced GTP-binding protein Mx1
OVA: ovalbumin
poly(I:C): polyinosinic:polycytidylic acid
RIG-I: retinoic acid-inducible gene 1
RV: rhinovirus
TLR3: toll-like receptor 3.
