# Supplementary material for: Azithromycin augments rhinovirus-induced IFNβ via cytosolic MDA5 in experimental models of asthma exacerbation
Source: Oncotarget. 2017 Mar 18;8(19):31601–11. doi: 10.18632/oncotarget.16364 (PMC5458233; doi:10.18632/oncotarget.16364)
Supplement: Supplementary file 1 [file oncotarget-08-31601-s001.pdf]

Azithromycin augments rhinovirus-induced IFNβ via cytosolic MDA5 in experimental models of asthma exacerbation

Supplementary Materials

SUPPLEMENTARY METHODS

Mouse bronchoalveolar lavage fluid analysis

Lavage of the lungs was performed with phosphate buffered saline (PBS) 24h after the final poly(I:C) exposure. Total cell count of the bronchoalveolar lavage fluid (BALF) supernatant was performed and total protein concentration was analysed by bicinchoninic acid (BCA) assay (Pierce, Thermo Scientific, Waltham, MA, USA). Furthermore lactate dehydrogenase levels were analysed in BALF according to the manufacturer's instructions (Roche Diagnostics, Bromma, Sweden).

RNA isolation and gene expression quantification by RT-qPCR

Total RNA from HBECs and mouse lung homogenates was extracted using a RNA extraction kit (Nucleospin® RNA II, Macherey-Nagel, Düren, Germany) and reverse transcribed to cDNA (Precision Nanoscript Reverse Transcription kit, PrimerDesign, Southampton, UK). Real-time quantitative PCR was run on an Mx3005P qPCR system (Stratagen, La Jolla, CA, USA) with standard cycling parameters. Primers were obtained from PrimerDesign or Qiagen (Sollentuna, Sweden). Primer sequences for primers provided by PrimerDesign are listed below:

IFNβ: TTACTTCATTAACAGACTTACAGGT (forward) and TACATAGCCATCGTCACTTAAAC (reverse),

HRV16: GAGAGGTTAAGAACTTGATTGAA (forward) and CTAATTTTGTTTGTGGTGATAGAG (reverse)

RIG-I: TTCTCTTGATGCGTCAGTGATA (forward) and CCGTGATTCCACTTTCCTGAA (reverse).

Analysis of samples was performed by the ΔΔCt method [1] and related to UBC/GAPDH expression for in vitro experiments and 18S rRNA for in vivo experiments. Groups were normalized to untreated control or RV16 for in vitro experiments when appropriate and HDM/saline/vehicle for in vivo experiments.

Quantification of interferon levels by ELISA

IFNβ levels were measured in cell supernatants of HBECs and in BALF of mice by ELISA according to the manufacturer's instructions (PBL Assay Science, Piscataway Township, NJ, USA).

Protein expression analysis of RIG-I like helicases by Western Blot

RIG-I and MDA5 protein expression was quantified by western blot analysis as described previously [2].

REFERENCES

1. Livak KJ, Schmittgen TD. Analysis of relative gene expression data using real-time quantitative PCR and the 2(-Delta Delta C(T)) Method. Methods. 2001; 25:402-8.
2. Menzel M, Akbarshahi H, Bjermer L, Uller L. Azithromycin induces anti-viral effects in cultured bronchial epithelial cells from COPD patients. Sci Rep. 2016; 6:28698.

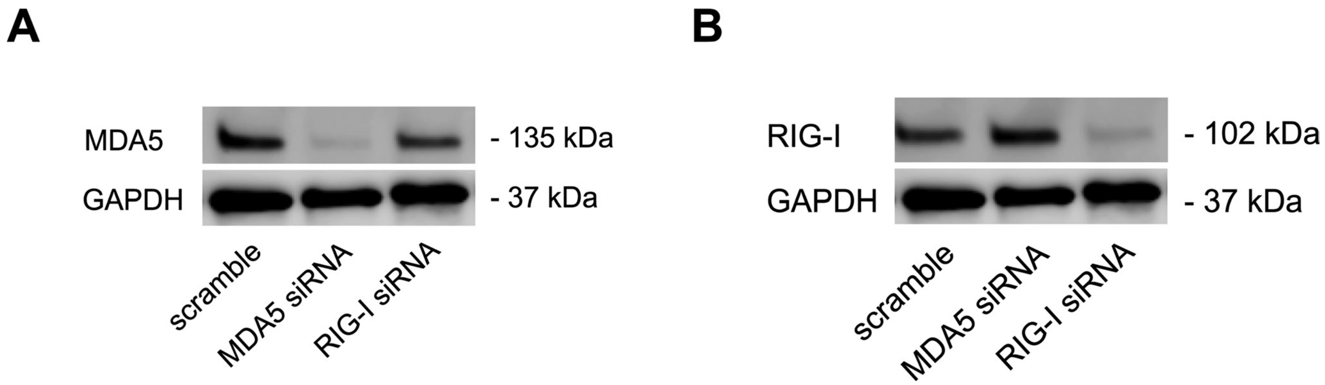

Supplementary Figure 1: Knockdown of MDA5 and RIG-I by siRNA in primary bronchial epithelial cells. HBECs from asthma patients were transfected with siRNA specific for MDA5 or RIG-I or non-specific siRNA (scramble). Cells were harvested 96h after siRNA transfection. A representative Western Blot of MDA5 (**A**) and RIG-I (**B**) protein is shown.
